# Fever education for caregivers in the emergency room (The FEVER study)–an interventional trial

**DOI:** 10.1038/s41390-024-03047-0

**Published:** 2024-01-25

**Authors:** Catherine J. Lynch, Maja Kuhar, Carol Blackburn, Michael J. Barrett

**Affiliations:** 1 Department of Paediatric Emergency Medicine, Children’s Health Ireland, Dublin, Ireland; 2 Women’s and Children’s Health, University College Dublin, Dublin, Ireland

## Abstract

**Background:**

Despite the vast majority of fevers representing benign self-limiting illnesses, caregiver anxiety regarding fever is high. Empowering caregivers with knowledge to safely and appropriately manage fever at home has the potential to reduce demands upon healthcare services.

**Aim:**

To improve caregiver knowledge about fever and its management in children via an educational intervention.

**Methods:**

Caregivers of children over 6 months presenting with fever to a Paediatric Emergency Department were recruited. A pre-intervention survey was completed to ascertain caregiver knowledge about fever and its management. The intervention of (i) an infographic about fever, with (ii) a short video on fever was viewed. A post-intervention survey re-assessed knowledge. The primary outcome was the correct definition of fever as a temperature ≥38 °C.

**Results:**

Caregivers (*n* = 51) who correctly defined fever increased from 41% (*n* = 21) pre-intervention to 94% (*n* = 48) post-intervention. There was a reduction in common misconceptions about fever, including a higher fever representing a more serious infection (76% vs. 8%). Caregivers reported they were less likely to seek emergency healthcare due to the height and nature of the fever alone.

**Conclusions:**

A simple brief educational intervention can rapidly increase caregiver knowledge about fever in children. There is a continuing need for clear, easily-accessible information for caregivers on this topic.

**Impact:**

- Parental knowledge about fever and how to manage it in their children is low.
- A simple brief educational intervention can significantly increase caregiver knowledge about fever.
- A combined written and audiovisual approach is effective and well-received by parents.
- Educating caregivers has the potential to improve the management of childhood fever at home and to reduce the burden on healthcare services, as well as reduce unpleasant hospital visits for children and their caregivers.

## Introduction

Fever is one of the most common concerns which results in presentation to the Emergency Department for children and their parents.^1,2^ Caregiver anxiety regarding fever is high, despite the vast majority of fevers representing a benign self-limiting illness.^3–6^ This can cause more frequent and urgent seeking of medical advice than is warranted.

Empowering caregivers with knowledge to safely and appropriately self-manage a child’s fever has the potential to reduce the burden on busy healthcare services, as well as reducing unpleasant, and most likely unnecessary, hospital visits for the children and their caregivers.^7,8^ We know that parents’ knowledge about fever in Ireland is suboptimal and that parents themselves have indicated the need for accessible and reliable information resources on the topic.^3,8,9^

Educational resources have been demonstrated to improve parents’ knowledge and management practices around fever as well as decreasing consultations with healthcare professionals for fever.^7,8,10–12^ This education can be delivered via a range of mediums, including verbal, written, and video resources. Whilst many of those accessing healthcare have indicated that verbal explanation from a healthcare professional is their preferred medium, we know that recall of this information is suboptimal.^13^ Between 40 and 80% of medical information is immediately forgotten by patients, and almost half of what is remembered is incorrect.^14,15^

Written information increases information recall and treatment adherence, but can be inaccessible to those with low education or literacy and non-native speakers.^10,11,16^ Visual information such as pictographs have been shown to significantly increase patient understanding in these instances.^17^ Multimedia interventions can also increase patient medical knowledge and recall.^11,12,18,19^ Whilst an individualised approach to health education would be ideal, this is challenging to achieve on a practical level, and hence a combination of multiple knowledge translation methods is likely to be of most benefit to the most patients.^7^

## Aim

The aim of this project was to improve caregiver knowledge about fever in children and its management via an educational intervention using both written and video content. The primary outcome was correct definition of fever as a temperature of greater than 38 degrees celsius.^3^ Secondary outcomes were improvements in understanding of management practices for fever, and reduction in common misconceptions about fever.

## Methods

### Study design

A prospective, single-centre, point-of-care education intervention study with pre- and post-test design with study exit within a single episode of care.

### Study sample

Caregivers with children over 6 months presenting to a tertiary urban Paediatric Emergency Department (PED) with fever were invited to participate. Potential participants were approached by a member of the research team, who were not involved in their care. Caregivers were approached whilst waiting to be seen in the ED. The normal flow and standards of their care were not affected by their participation in the study. An information leaflet about the study was provided, and consent was obtained anonymously on each survey completed.

### Information leaflet

An information leaflet was designed by the study team (Appendix 1) with the included information based on previous similar studies with proven efficacy.^10^ The National Adult Literacy Agency (NALA) checklist for Plain English was used when considering the text and layout of the leaflet. The design was original, with images to support the information in each paragraph, and a question-answer approach as recommended by NALA to aid knowledge translation. Input was sought from doctors, nurses and healthcare assistants from the multi-disciplinary team in the PED, as well as non-medical parents, prior to finalising the leaflet.

### Video

A video script was produced by the research team, covering the same information as the information leaflet. This was recorded using a smartphone, with a clinician from the Emergency Department (also a member of the research team) relaying the information. The video was edited, and a few words of text were added in the top right-hand corner to emphasise the most important points. The final version was just over 3 minutes long in total. This was uploaded onto the Department’s *Vimeo* account, and a QR code link to the video was created and added to the leaflet above.^20^

### Intervention

A pre-intervention questionnaire (Appendix 2) was completed by the caregiver to ascertain their knowledge about fever and its management in children. The questionnaire consisted of eight questions about fever, followed by three questions about demographics and one regarding information sources, and was anonymised.

Caregivers were then given the information leaflet about fever, which included a QR code link to the video which could be accessed via their smartphone.

A post-intervention questionnaire (Appendix 3) was then completed by caregivers to re-assess their knowledge about fever. This included the same eight questions about fever, followed by questions about satisfaction with and feedback about the leaflet and video interventions.

### Study outcomes

The primary outcome was the correct definition of fever as a temperature ≥38 °C. Secondary outcomes were improvements in management practices about fever (medication use and fever-reduction techniques such as tepid sponging, use of fans, and removal of clothing), reasons for seeking healthcare, and common misconceptions about fever in children.

### Sample size

A sample size calculation was based on results from a previous population study in Ireland which showed that 37% of parents correctly identified the temperature at which their child could be said to have a fever. We anticipated this to increase to 69% after the intervention – a minimum clinically important difference of 32. With 80% power and a type one error rate of alpha = 0.05, a minimum sample size of 17 was required. The target minimum sample size was 17 within 3 months (due to researcher availability) however a sample size of 47 was desirable. A sample size of 47, with a type 1 error rate of alpha = 0.05, would achieve 95% power.

### Data analysis

Data was compiled into and processed using an Excel spreadsheet. Associations between categorical variables were assessed using Pearson’s Chi-squared test. *P* values of less than 0.05 were considered statistically significant.

## Results

### Participation and demographics

A convenience sample was recruited between February and April 2023, based on the availability of the research team. 51 caregivers participated in the study, and all completed both pre- and post-intervention questionnaires. The demographics of caregivers are provided in Table 1. The vast majority were female, with an average age of 32 years. The mean number of children per caregiver was two.

**Table 1:** Caregiver demographics.

Caregiver demographics		*n* = 51
Age (yrs)	Range	19–42
	Mean	32.5
	Median	32
Gender *n*, (%)	Male	9 (18)
	Female	42 (82)
	Other	0 (0)
No. of children	Range	1–4
	Mean	2

### Primary outcome

Caregivers (*n* = 51) who correctly identified fever as a temperature above 38.0 °C increased from 41% (*n* = 21) pre-intervention to 94% (*n* = 48) post-intervention (*p* = <0.0001)(Table 2).

**Table 2:** Primary outcome.

	Pre-intervention	Post-intervention	*p* value*
Caregivers identifying fever as ≥38 °C, *n*, (%)	21 (41)	48 (94)	<0.0001

*Pearson’s Chi-squared test.

### Secondary outcomes

Secondary outcomes are summarised in Table 3.

**Table 3:** Secondary outcomes.

	Pre-intervention	Post-intervention	*p* value*
Fever reduction measures			
Cold compress/tepid sponging, n, (%)	33 (65)	8 (16)	<0.0001
Fan, *n*, (%)	6 (12)	0 (0)	0.027^†^
Remove all clothes, *n*, (%)	34 (67)	9 (18)	<0.0001
Medication use			
Always, regardless of distress, *n*, (%)	40 (78)	11 (22)	<0.0001
Only if distressed, n, (%)	11 (22)	40 (78)	<0.0001
Reasons for seeking healthcare			
Height of fever, *n*, (%)	22 (43)	3 (6)	<0.0001
Antipyretics not reducing fever, *n*, (%)	35 (69)	6 (12)	<0.0001
Child’s behaviour abnormal, *n*, (%)	38 (75)	46 (90)	0.038
Concern re dehydration, *n*, (%)	29 (57)	38 (75)	0.061
Parental instinct that child is very unwell, *n*, (%)	38 (75)	42 (82)	0.336
Common misconceptions			
Higher fever represents more serious illness, n, (%)	39 (76)	4 (8)	<0.0001
Temperature not normalising with antipyretics indicates more serious illness, n, (%)	47 (92)	4 (8)	<0.0001

^*^Pearson’s Chi-squared test, unless otherwise indicated.

^†^Fisher’s exact test.

#### Fever-reducing measures

There was a reduction in parents who would use fever-reducing measures which are not recommended. Those who reported they would sponge their child’s forehead with cool water reduced from 65% (*n* = 33) to 16% (n = 8) following the intervention (*p* = <0.0001). Those who reported they would use a fan to reduce their child’s temperature fell from 12% (*n* = 6) to 0% (*n* = 0). Finally, those who would strip their child naked in an effort to manage their child’s fever reduced from 67% (*n* = 34) to 18% (*n* = 9) (*p* = <0.0001).

#### Use of medication

Prior to the intervention 78% (*n* = 40) of caregivers indicated that they would “always” use antipyretic medication in the event of their child having a fever, compared to 22% (*n* = 11) of caregivers following the intervention (*p* = <0.0001). Caregivers who reported they would give antipyretic medication only if their child was distressed with a fever increased from 22% (*n* = 11) to 78% (*n* = 40) (*p* = <0.0001).

#### Healthcare seeking behaviours

Caregivers who reported that they would seek a healthcare consultation due to the height or nature of the fever alone reduced significantly following the intervention. Those who would see a doctor due to the height of the fever fell from 43% (*n* = 22) to 6% (*n* = 3) (*p* = <0.0001), whilst those who would present due to the fever not coming down with antipyretics reduced from 69% (*n* = 35) to 12% (*n* = 6) (*p* = <0.0001). Conversely, there was an increase in caregivers who reported that they would seek medical advice based on their child’s behaviour being abnormal (*p* = 0.038), and an increase based on concerns regarding dehydration, or if their instinct as a parent was that their child was very unwell, although these did not reach statistical significance.

#### Common misconceptions

There was a reduction in common misconceptions about fever. Caregivers who believed that the temperature not returning to normal after antipyretics representing more serious infection fell from 92% (*n* = 47) pre-intervention to 8% (*n* = 4) post-intervention (*p* = <0.0001). Similarly, caregivers who believed that a higher fever represented a more serious illness fell from 76% (*n* = 39) to 8% (*n* = 4) following the intervention (*p* = <0.0001).

#### Satisfaction with the leaflet and video

Caregivers reported that they found both the leaflet and video helpful (100% and 98% respectively). Comments about the leaflet included: “brilliant information”, “very useful”, “nicely laid out” and “great for new parents”. Parents suggested further improvements to the resources, including discussing other topics such as rigors and sepsis, and having an online version of the leaflet for future reference.

## Discussion

This point of care intervention study revealed that an information leaflet and video can significantly increase caregiver knowledge about fever and its management practises. Pre-intervention, 41% of caregivers correctly identified the temperature at which fever is said to be present. This is consistent with a previous study in which 37% of parents selected 38 degrees as temperature to define fever in their child.^3^ Subsequent to reading the leaflet and watching the video, 94% of caregivers correctly identified fever, representing a profound treatment effect (*p* = <0.0001).

Post-intervention, caregivers were less likely to engage in fever-reduction techniques which are of no benefit to their children, such as the use of tepid sponging or fans, and less likely to use antipyretic medication unless indicated by distress in their child.

Parents have highlighted the need for information resources regarding their children’s health.^3,8,9^ In line with previous studies, this study shows that our information resources can increase parental knowledge, and suggests that they can empower parents to make more informed choices regarding the management of their children’s febrile illnesses in the future.^7,10–12,19^

Whilst it is widely accepted that increasing parental health knowledge and health literacy improves outcomes for their children,^21^ the best means by which to translate this knowledge is less certain. Previous studies have shown the effectiveness of written information on parental knowledge increase and retention,^7,10^ whilst further studies have suggested the benefits of visual and multimedia information on patient understanding and recall of information.^11,12,19^ Our study used a combination of written, visual and aural information and shows that this is effective in achieving an improvement in participants’ knowledge.

Following the intervention in this study, caregivers were less likely to believe that the height or response of a fever to medication was indicative of the severity of the underlying disease, and reported they would be less likely to seek urgent medical advice purely due to the height or nature of the fever itself. This is in keeping with previous studies which have shown a reduction in healthcare service use following educational interventions.^7^ The authors believe this is a highly relevant finding for healthcare professionals working in paediatric unscheduled care, where annually increasing attendances of children who do not require the services of an emergency department has been observed and described.^22^ While not the primary outcome measure, the finding suggests that a short, low-cost, educational intervention has the potential to reduce unnecessary unscheduled care attendances to emergency services if delivered to parents opportunely.

### Strengths and Limitations

Strengths of our study include its clearly defined aim, intervention and outcome measures, which were able to clearly demonstrate the benefits of our intervention. The desirable sample size was recruited.

A limitation of our study is that the post-intervention questionnaire was completed immediately after the intervention was delivered. This does not allow for assessment of retention of knowledge by participants which is an important part of the education process. Our study was a single-centre study which places some limitations on the applicability of its results in different settings. However, it was reassuring to see the concordance between pre-intervention knowledge rates about fever and those from other similar studies.^3^ A convenience sample of parents, who elected to participate, raises the potential for self-selection of particular socio-economic groups rather than being a true representation of all those presenting to our PED. In the future, research partnership (rather than simple consultation) with co-design and co-production of these resources would be preferable, in line with best practice, and will be a focus of future work.

## Conclusions

A simple brief educational intervention can rapidly increase caregiver knowledge about fever in children. There is a continuing need for easily-accessible, well signposted and clear information for caregivers on this topic.

### Supplementary information

Appendix 1

Appendix 2

Appendix 3

## Data Availability

The datasets generated during this study are available from the corresponding author on reasonable request.
