# Supplementary material for: Fever education for caregivers in the emergency room (The FEVER study)–an interventional trial
Source: Pediatr Res. 2024 Jan 25;96(1):172–6. doi: 10.1038/s41390-024-03047-0 (PMC11257936; doi:10.1038/s41390-024-03047-0)
Supplement: Supplementary file 1 — Appendix 1 [file 41390_2024_3047_MOESM1_ESM.pdf]

FEVER IN CHILDREN

aged > 6 months

WHAT IS A FEVER?

A raised body temperature over 38 degrees.
It is best to use a digital in-ear thermometer.

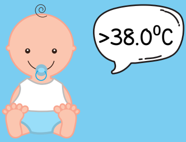

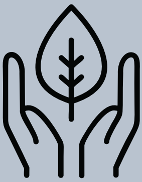

WHY DOES MY CHILD HAVE A FEVER?

Fever is the body's **NATURAL** response to infection.
It helps the body fight infection and will not damage your child

WHAT IF IT'S VERY HIGH?

The height of the fever has **no correlation** with how unwell your child is.
Minor illnesses may cause fevers of 40°C. Serious illnesses may cause only a mild fever.

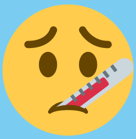

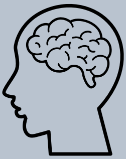

BUT WHAT ABOUT FEBRILE SEIZURES?

The risk of convulsions is **not** just related to the height of the fever.
A child's genes play a much bigger role.

SO THEN HOW DO I KNOW HOW SICK MY CHILD IS?

Your child's behaviour, alertness, energy and responsiveness are the most important indicators of how unwell your child is.

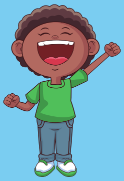

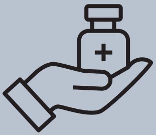

USING MEDICINES

Paracetamol (Calpol®) and/or Ibuprofen (Nurofen®) can be used to treat the aches and pains that come with fever.
If your child is comfortable, you do not have to treat the fever.

SHOULD I WORRY IF THE FEVER DOESN'T COME DOWN?

No, this does **not** mean the infection is more serious.
Your child's behaviour and hydration are much more important.

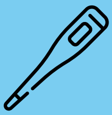

WHEN TO SEEK URGENT MEDICAL ATTENTION

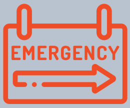

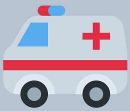

Come to the emergency department or call an ambulance if your child has:

- Very fast breathing
- Excessive drowsiness or is difficult to wake
- Convulsions
- Dehydration or has not passed urine for over 12 hours
- Rash that does not go away when you press it
- Abnormally cold to touch
- Your instinct as a parent is that your child is very unwell.

MANAGING FEVER AT HOME

DO

- Ensure your child drinks plenty of fluids
- Treat aches and pains
- Dress them comfortably – remove excess blankets or outer clothes if needs be.
- Check on your child regularly, and during the night

DON'T

- Sponge your child with water or use cold compresses on the forehead
- Use a fan
- Remove all your child’s clothes
- Wake a sleeping child just to give them medicine

WHERE CAN I FIND OUT MORE?

undertheweather.ie
mychild.ie

Our video - please scan the QR code :
