# Supplementary material for: Fever education for caregivers in the emergency room (The FEVER study)–an interventional trial
Source: Pediatr Res. 2024 Jan 25;96(1):172–6. doi: 10.1038/s41390-024-03047-0 (PMC11257936; doi:10.1038/s41390-024-03047-0)
Supplement: Supplementary file 2 — Appendix 2 [file 41390_2024_3047_MOESM2_ESM.pdf]

Children's Health Ireland

The FEVER Study

Questionnaire 1

Dear Participant,

Thank you for taking the time to complete this survey. Prior to proceeding, your consent is required. Please read the following and tick the check boxes if you agree with the following statements:

Please tick the box next to each statement if you understand and agree	Agree
I have read and understand the study Participant Information Leaflet (PIL).	
I understand that this survey is intended to be anonymous and should not identify any particular individual.	
I understand that should I provide my name or other identifiable information; my responses may be linked to me.	
I understand that if I include any identifiable information, these will be anonymised by the researchers.	
I understand that on completion of this survey I am providing consent for the use of this data.	
I understand that on completion of this survey, as everything is anonymised it will not be possible to have my specific responses removed from the pooled data.	

There are 7 short questions about fever, and 5 short questions about yourself. Please tick the appropriate box(es) for each question:

1. At what temperature would you consider your child to have a fever?

- ☐ Above 36.5°C
- ☐ Above 37.0°C
- ☐ Above 37.5°C
- ☐ Above 38.0°C
- ☐ Above 38.5°C
- ☐ Above 39.0°C
- ☐ Above 39.5°C
- ☐ Above 40.0°C

2. How do you measure your child's temperature?

- ☐ Under-the-arm digital thermometer
- ☐ In-the-ear digital thermometer
- ☐ Infrared forehead thermometer
- ☐ Other ________________________________

3. Would you give your child a medication such as Paracetamol (Calpol®) or Ibuprofen (Nurofen®) if they had a fever?

- ☐ Yes, always
- ☐ Yes, but only if they were distressed
- ☐ No

Thank you very much for your time, help and contribution to this study!

4. Why would you use Paracetamol (Calpol®) or Ibuprofen (Nurofen®) when your child has a fever?
   - ☐ To bring the fever down
   - ☐ To relieve the aches and pains associated with fever
   - ☐ Other – please explain:
     ____

5. Does a fever of 40°C mean your child has a more serious illness than if they have a fever of 38°C?
   - ☐ Yes
   - ☐ No

6. If your child's temperature does not return to normal after giving Paracetamol (Calpol®) or Ibuprofen (Nurofen®), does this mean the infection they have is more serious?
   - ☐ Yes
   - ☐ No

7. Which of the following are additional ways you could help manage fever in your child at home? (tick all that apply)
   - ☐ Remove excess blankets
   - ☐ Sponge their forehead with cool water
   - ☐ Use a fan
   - ☐ Remove all their clothes down to nappy/underwear
   - ☐ Ensure they drink plenty of fluids

8. When would you see a doctor when your child has a fever?
   - ☐ If the fever is very high
   - ☐ If the fever does not come down with Calpol®/Nurofen®
   - ☐ If my child appears dehydrated
   - ☐ If my child's behaviour is abnormal (e.g., difficult to wake or excessively drowsy)
   - ☐ If my instinct as a parent is that my child is very unwell

9. What is your age?

10. What is your gender?
    - ☐ Male
    - ☐ Female
    - ☐ Other

11. How many children do you have? What age are they?

12. Where do you go to get healthcare information for your children?
    - ☐ Family member
    - ☐ Google
    - ☐ HSE website
    - ☐ GP
    - ☐ Social media
    - ☐ Books (eg My Child book)
    - ☐ Other specific website:____
    - ☐ Other: please explain: ____

Thank you very much for your time, help and contribution to this study!
